# Strong convergence theorems by hybrid and shrinking projection methods for sums of two monotone operators

**DOI:** 10.1186/s13660-017-1338-7

**Published:** 2017-04-11

**Authors:** Tadchai Yuying, Somyot Plubtieng

**Affiliations:** 1 Department of Mathematics, Faculty of Science, Naresuan University, Phitsanulok, 65000 Thailand; 2 Research center for Academic Excellence in Mathematics, Naresuan University, Phitsanulok, 65000 Thailand

**Keywords:** hybrid projection methods, shrinking projection methods, monotone operators and resolvent

## Abstract

In this paper, we introduce two iterative algorithms for finding the solution of the sum of two monotone operators by using hybrid projection methods and shrinking projection methods. Under some suitable conditions, we prove strong convergence theorems of such sequences to the solution of the sum of an inverse-strongly monotone and a maximal monotone operator. Finally, we present a numerical result of our algorithm which is defined by the hybrid method.

## Introduction

The monotone inclusion problem is very important in many areas, such as convex optimization and monotone variational inequalities, for instance. Splitting methods are very important because many nonlinear problems arising in applied areas such as signal processing, machine learning and image recovery which mathematically modeled as a nonlinear operator equation which this operator can be consider as the sum of two nonlinear operators. The problem is finding a zero point of the sum of two monotone operators; that is,

1 $$ \text{find }z\in H \text{ such that }0\in(A+B)z, $$

where *A* is a monotone operator and *B* is a multi-valued maximal monotone operator. The set of solutions of (1) is denoted by $(A+B)^{-1}(0)$. We know that the problem (1) included many problems; see for more details [1–8] and the references therein. In fact, we can formulate the initial value problem of the evolution equation $0 \in Tu + \frac{\partial u}{\partial t}$, $u = u(0)$, as the problem (1) where the governing maximal monotone *T* is of the form $T = A + B$ (see [6] and the references therein). The methods for solving the problem (1) have been studied extensively by many authors (see [4, 6] and [9]).

In 1997, Moudafi and Thera [10] introduced the iterative algorithm for the problem (1) where the operator *B* is maximal monotone and *A* is (single-valued) Lipschitz continuous and strongly monotone such as the iterative algorithm

2 $$ \textstyle\begin{cases} x_{n} = J_{\lambda}^{B}w_{n},\\ w_{n+1}=sw_{n}+(1-s)x_{n}-\lambda(1-s)Ax_{n}, \end{cases} $$

with fixed $s\in(0,1)$ and under certain conditions. They found that the sequence $\{x_{n}\}$ defined by (2) converges weakly to elements in $(A+B)^{-1}(0)$.

On the other hand, Nakago and Takahashi [11] introduced an iterative hybrid projection method and proved the strong convergence theorems for finding a solution of a maximal monotone case as follows:

3 $$ \textstyle\begin{cases} x_{0}=x \in H, \\ y_{n}=J_{r_{n}}(x_{n}+f_{n}), \\ C_{n}=\{z\in H: \Vert y_{n}-z \Vert \leq \Vert x_{n}+f_{n}-z \Vert \}, \\ Q_{n}=\{z\in H: \langle x_{n}-z,x_{0}-x_{n}\rangle\geq0\}, \\ x_{n+1}=P_{C_{n}\cap Q_{n}}(x_{0}), \end{cases} $$

for every $n\in\mathbb{N}\cup\{0\}$, where $r_{n}\subset(0, \infty)$. They proved that if $\liminf_{n\rightarrow\infty}r_{n}>0$ and $\lim_{n\rightarrow\infty} \Vert f_{n} \Vert =0$, then $x_{n}\rightarrow z_{0}=P_{A^{-1}(0)}(x_{0})$. Furthermore, many authors have introduced the hybrid projection algorithm for finding the zero point of maximal monotones such as [12] and other references. Recently, Qiao-Li Dong *et al.* [13] introduced a new hybrid projection algorithm for finding a fixed point of nonexpansive mappings. Under suitable assumptions, they proved that such sequence converge strongly to a solution of fixed point *T*. Moreover, by using a shrinking projection method, Takahashi et al. [14] introduced a new algorithm and proved strong convergence theorems for finding a common fixed point of families of nonexpansive mappings.

In this paper motivated by the iterative schemes considered in the present paper, we will introduce two iterative algorithms for finding zero points of the sum of an inverse-strongly monotone and a maximal monotone operator by using hybrid projection methods and shrinking projection methods. Under some suitable conditions, we obtained strong convergence theorems of the iterative sequences generated by the our algorithms. The organization of this paper is as follows: Section 2, we recall some definitions and lemmas. Section 3, we prove a strong convergence theorem by using hybrid projection methods. Section 4, we prove a strong convergence theorem by using shrinking projection methods. Section 5, we report a numerical example which indicate that the hybrid projection method is effective.

## Preliminaries

In this paper, we let *C* be a nonempty closed convex subset of a real Hilbert space *H*. Denote $P_{C}(\cdot)$ is the metric projection on *C*. It is well known that $z=P_{C}(x)$ if

$$\langle x-z,z-y\rangle\geq0\quad\text{for all }y\in C. $$

Moreover, we also note that

$$\bigl\Vert P_{C}(x)-P_{C}(y) \bigr\Vert \leq \Vert x-y \Vert \quad\text{for all }x,y\in H $$

and

$$\bigl\Vert P_{C}(x)-x \bigr\Vert \leq \Vert x-y \Vert \quad \text{for all }y \in C $$

(see also [15]). We say that $A: C \to H$ is a monotone operator if

$$\langle Ax-Ay,x-y\rangle\geq0\quad\text{for all }x,y \in C, $$

and the operator $A: C \to H$ is inverse-strongly monotone if there is $\alpha>0$ such that

$$\langle Ax-Ay,x-y\rangle\geq\alpha \Vert Ax-Ay \Vert ^{2}\quad \text{for all }x,y \in C. $$

For this case, the operator *A* is called *α*-inverse-strongly monotone. It is easy to see that every inverse-strongly monotone is monotone and continuous. Recall that $B:H \to2^{H}$ is a set-valued operator. Then the operator *B* is monotone if $\langle x_{1}-x_{2},z_{1}-z_{2}\rangle\geq0$ whenever $z_{1}\in Bx_{1}$ and $z_{2}\in Bx_{2}$. A monotone operator *B* is maximal if for any $(x,z)\in H\times H$ such that $\langle x-y,z-w\rangle\geq0$ for all $(y,w)\in\operatorname{Graph}B$ implies $z\in Bx$. Let *B* be a maximal monotone operator and $r>0$. Then we can define the resolvent $J_{r}: R(I+rB)\to D(B)$ by $J_{r}=(I+rB)^{-1}$ where $D(B)$ is the domain of *B*. We know that $J_{r}$ is nonexpensive and we can study the other properties in [15–17].

### Lemma 2.1

[18]

*Let* *C* *be a closed convex subset of a real Hilbert space* *H*, $x\in H$. *and* $z= P_{C}x$. *If* $\{x_{n}\}$ *is a sequence in* *C* *such that* $\omega _{w}(x_{n})\subset C$ *and*

$$\Vert x_{n}-x \Vert \leq \Vert x-z \Vert , $$

*for all* $n\geq1$, *then the sequence* $\{x_{n}\}$ *converges strongly to a point* *z*.

### Lemma 2.2

[13]

*Let* $\{\alpha_{n}\}$ *and* $\{\beta_{n}\}$ *be nonnegative real sequences*, $a\in[0,1)$ *and* $b\in\mathbb{R}^{+}$. *Assume that*, *for any* $n\in\mathbb{N}$,

$$\alpha_{n+1}\leq a\alpha_{n}+b\beta_{n}. $$

*If* $\sum_{n=1}^{\infty}\beta_{n}< +\infty$, *then* $\lim_{n\rightarrow\infty}\alpha_{n}=0$.

### Lemma 2.3

[18]

*Let* *C* *be a closed convex subset a real Hilbert space* *H*, *and* $x,y,z\in H$. *Then*, *for given* $a\in\mathbb{R}$, *the set*

$$U=\bigl\{ v\in C: \Vert y-v \Vert ^{2}\leq \Vert x-v \Vert ^{2}+\langle z,v\rangle+a\bigr\} $$

*is convex and closed*.

### Lemma 2.4

[19]

*Let* *C* *be a nonempty closed convex subset of a real Hilbert space* *H*, *and* $A: C\to H$ *an operator*. *If* $B:H\to2^{H}$ *is a maximal monotone operator*, *then*

$$F\bigl(J_{r}(I-rA)\bigr)=(A+B)^{-1}(0). $$

## Hybrid projection methods

In this section, we introduce a new iterative hybrid projection method and prove a strong convergence theorem for finding a solution of the sum of an *α*-inverse-strongly monotone (single-value) operator and a maximal monotone (multi-valued) operator.

### Theorem 3.1

*Let* *C* *be a nonempty closed convex subset of a real Hilbert space* *H*. *Suppose that* $A: C \to H$ *is an* *α*-*inverse*-*strongly monotone operator and let* $B: H\to 2^{H}$ *be a maximal monotone operator with* $D(B)\subseteq C$ *and* $(A+B)^{-1}(0) \neq\emptyset$. *Define a sequence* $\{x_{n}\}$ *by the algorithm*

4 $$ \textstyle\begin{cases} x_{0}, z_{0}\in C, \\ y_{n}=\alpha_{n}z_{n}+(1-\alpha_{n})x_{n}, \\ z_{n+1}=J_{r_{n}}(y_{n}-r_{n}Ay_{n}), \\ C_{n}=\{z\in C: \Vert z_{n+1}-z \Vert ^{2}\leq\alpha _{n} \Vert z_{n}-z \Vert ^{2}+(1-\alpha_{n}) \Vert x_{n}-z \Vert ^{2}\}, \\ Q_{n}=\{z\in C: \langle x_{n}-z,x_{0}-x_{n}\rangle\geq0\}, \\ x_{n+1}=P_{C_{n}\cap Q_{n}}(x_{0}), \end{cases} $$

*for all* $n\in\mathbb{N}\cup\{0\}$, *where* $J_{r_{n}}=(I+r_{n}B)^{-1}$, $\{\alpha_{n}\}$ *and* $\{r_{n}\}$ *are sequences of positive real numbers with* $0\leq\alpha_{n}\leq\beta$ *for some* $\beta\in[0,\frac{1}{2})$ *and* $0< r_{n}\leq2\alpha$. *Then the sequence* $\{x_{n}\}$ *converges strongly to a point* $p=P_{(A+B)^{-1}(0)}(x_{0})$.

### Proof

From Lemma 2.3, we see that $C_{n}$ is closed convex for every $n\in\mathbb{N}\cup\{0\}$. First, we show that $(A+B)^{-1}(0)\subset C_{n}$ for all $n\in\mathbb{N}\cup\{0\}$. Since $A: C \to H$ is an *α*-inverse-strongly monotone operator, we have $I-r_{n}A$ is nonexpensive. Indeed,

$$\begin{aligned} \bigl\Vert (I-r_{n}A)x-(I-r_{n}A)y \bigr\Vert ^{2} =& \bigl\Vert (x-y)-r_{n}(Ax-Ay) \bigr\Vert ^{2} \\ =& \Vert x-y \Vert ^{2}-2r_{n}\langle x-y,Ax-Ay\rangle +r_{n}^{2} \Vert Ax-Ay \Vert ^{2} \\ \leq& \Vert x-y \Vert ^{2}- r_{n}(2 \alpha-r_{n}) \Vert Ax-Ay \Vert ^{2} \\ \leq& \Vert x-y \Vert ^{2}. \end{aligned}$$

Let $n\in\mathbb{N}\cup\{0\}$ and $w \in(A+B)^{-1}(0)$. Thus, we have

$$\begin{aligned} \Vert z_{n+1}-w \Vert ^{2} =& \bigl\Vert J_{r_{n}}(y_{n}-r_{n}Ay_{n})-J_{r_{n}}(w-r_{n}A w) \bigr\Vert ^{2} \\ \leq& \bigl\Vert (y_{n}-r_{n}Ay_{n})-(w-r_{n}A w) \bigr\Vert ^{2} \\ \leq& \Vert y_{n}-w \Vert ^{2} \\ =& \bigl\Vert \alpha_{n}z_{n}+(1-\alpha_{n})x_{n}-w \bigr\Vert ^{2} \\ \leq& \alpha_{n} \Vert z_{n}-w \Vert ^{2}+(1-\alpha_{n}) \Vert x_{n}-w \Vert ^{2}. \end{aligned}$$

This implies that $w \in C_{n}$ for all $n\in\mathbb{N}\cup\{0\}$ and hence

5 $$ (A+B)^{-1}(0)\subset C_{n}, $$

for all $n\in\mathbb{N}\cup\{0\}$. Next, we prove that $(A+B)^{-1}(0)\subset Q_{n}$ for all $n\in\mathbb{N}\cup\{0\}$ by the mathematical induction. For $n=0$, we note that

$$(A+B)^{-1}(0)\subset C= Q_{0}. $$

Suppose that $(A+B)^{-1}(0)\subset Q_{k}$ for some $k\in\mathbb{N}$. Since $C_{k}\cap Q_{k}$ is closed and convex, we can define

$$x_{k+1}=P_{C_{k}\cap Q_{k}}(x_{0}). $$

It follows that

$$\langle x_{k+1}-z, x_{0}-x_{k+1}\rangle\geq0 \quad \text{for all }z\in C_{k}\cap Q_{k}. $$

From $(A+B)^{-1}(0)\subset C_{k}\cap Q_{k}$, we see that

$$(A+B)^{-1}(0)\subset Q_{k+1}. $$

Therefore

6 $$ (A+B)^{-1}(0)\subset Q_{n}, $$

for all $n\in\mathbb{N}\cup\{0\}$. Combining the inequalities (5) and (6), it follows that $\{x_{n}\}$ is well defined.

Since $(A+B)^{-1}(0)$ is a nonempty closed convex set, there is a unique element $p\in(A+B)^{-1}(0)$ such that

$$p=P_{(A+B)^{-1}(0)}(x_{0}). $$

From $x_{n}=P_{Q_{n}}(x_{0})$, we have

$$\Vert x_{n}-x_{0} \Vert \leq \Vert q-x_{0} \Vert \quad\text{for all }q\in Q_{n}. $$

Due to $p\in(A+B)^{-1}(0)\subset Q_{n}$, we have

7 $$ \Vert x_{n}-x_{0} \Vert \leq \Vert p-x_{0} \Vert , $$

for any $n\in\mathbb{N}\cup\{0\}$. It follows that $\{x_{n}\}$ is bounded. As $x_{n+1}\in C_{n}\cap Q_{n}\subset Q_{n}$, we have

$$\langle x_{n}-x_{n+1},x_{0}-x_{n} \rangle\geq0, $$

and hence

8 $$\begin{aligned} \Vert x_{n+1}-x_{n} \Vert ^{2} =& \bigl\Vert (x_{n+1}-x_{0})-(x_{n}-x_{0}) \bigr\Vert ^{2} \\ =& \Vert x_{n+1}-x_{0} \Vert ^{2}- \Vert x_{n}-x_{0} \Vert ^{2}-2\langle x_{n+1}-x_{n},x_{n}-x_{0}\rangle \\ \leq& \Vert x_{n+1}-x_{0} \Vert ^{2}- \Vert x_{n}-x_{0} \Vert ^{2}. \end{aligned}$$

By (7) and (8), we have

$$\begin{aligned} \sum_{n=1}^{N} \Vert x_{n+1}-x_{n} \Vert ^{2} \leq& \sum_{n=1}^{N} \bigl( \Vert x_{n+1}-x_{0} \Vert ^{2}- \Vert x_{n}-x_{0} \Vert ^{2}\bigr) \\ =& \Vert x_{N+1}-x_{0} \Vert ^{2}- \Vert x_{1}-x_{0} \Vert ^{2} \\ \leq& \Vert q-x_{0} \Vert ^{2}- \Vert x_{1}-x_{0} \Vert ^{2}. \end{aligned}$$

Since *N* is arbitrary, $\sum_{n=1}^{\infty} \Vert x_{n+1}-x_{n} \Vert ^{2}$ is convergent and hence

9 $$ \Vert x_{n+1}-x_{n} \Vert \rightarrow0 \quad \text{as } n\rightarrow\infty. $$

Since $x_{n+1}\in C_{n}\cap Q_{n}\subset C_{n}$, we have

$$\begin{aligned} \Vert z_{n+1}-x_{n+1} \Vert ^{2} \leq& \alpha_{n} \Vert z_{n}-x_{n+1} \Vert ^{2}+(1-\alpha _{n}) \Vert x_{n}-x_{n+1} \Vert ^{2} \\ =& \alpha_{n}\bigl( \Vert z_{n}-x_{n} \Vert ^{2}+2\langle z_{n}-x_{n},x_{n}-x_{n+1} \rangle+ \Vert x_{n}-x_{n+1} \Vert ^{2}\bigr) \\ & {}+(1-\alpha_{n}) \Vert x_{n}-x_{n+1} \Vert ^{2} \\ \leq& 2\alpha_{n}\bigl( \Vert z_{n}-x_{n} \Vert ^{2}+ \Vert x_{n}-x_{n+1} \Vert ^{2}\bigr) +(1-\alpha_{n}) \Vert x_{n}-x_{n+1} \Vert ^{2} \\ =& 2\alpha_{n} \Vert z_{n}-x_{n} \Vert ^{2}+ (1+\alpha _{n}) \Vert x_{n}-x_{n+1} \Vert ^{2} \\ \leq& 2\beta \Vert z_{n}-x_{n} \Vert ^{2}+ 2 \Vert x_{n}-x_{n+1} \Vert ^{2}, \end{aligned}$$

for all $n\in\mathbb{N}$. By Lemma 2.2 and $\beta\in [0,\frac{1}{2})$, we get

10 $$ \Vert z_{n}-x_{n} \Vert \rightarrow0 \quad \text{as } n\rightarrow\infty. $$

In fact, since $\Vert z_{n+1}-x_{n} \Vert \leq \Vert z_{n+1}-x_{n+1} \Vert + \Vert x_{n+1}-x_{n} \Vert $, for all $n\in\mathbb{N}$, it follows by (9) and (10) that

11 $$ \Vert z_{n+1}-x_{n} \Vert \rightarrow0 \quad \text{as } n\rightarrow\infty. $$

Note that

$$\begin{aligned} \Vert x_{n}-y_{n} \Vert =& \bigl\Vert x_{n}-\alpha _{n}z_{n}-(1-\alpha_{n})x_{n} \bigr\Vert \\ =& \alpha_{n} \Vert x_{n}-z_{n} \Vert \\ \leq& \beta \Vert x_{n}-z_{n} \Vert , \end{aligned}$$

for all $n\in\mathbb{N}$. Thus, we see that

12 $$ \Vert x_{n}-y_{n} \Vert \rightarrow0 \quad \text{as } n\rightarrow\infty. $$

Moreover, we note that

$$\begin{gathered} \bigl\Vert J_{r_{n}}(I-r_{n}A)x_{n}-x_{n} \bigr\Vert \\ \quad \leq \bigl\Vert J_{r_{n}}(I-r_{n}A)x_{n}-J_{r_{n}}(I-r_{n}A)y_{n} \bigr\Vert + \bigl\Vert J_{r_{n}}(I-r_{n}A)y_{n}-z_{n+1} \bigr\Vert + \Vert z_{n+1}-x_{n} \Vert \\ \quad \leq \Vert x_{n}-y_{n} \Vert + \Vert z_{n+1}-x_{n} \Vert , \end{gathered} $$

for all $n\in\mathbb{N}$. By (11) and (12), we see that

13 $$ \bigl\Vert J_{r_{n}}(I-r_{n}A)x_{n}-x_{n} \bigr\Vert \rightarrow0 \quad \text{as } n\rightarrow\infty. $$

From (13), it follows by the demiclosed principle (see [20]) that

$$\omega_{w}(x_{n})\subset F\bigl(J_{r_{n}}(I-r_{n}A) \bigr)=(A+B)^{-1}(0). $$

Hence by Lemma 2.1 and (7), we can conclude that the sequence $\{x_{n}\}$ converges strongly to $p=P_{(A+B)^{-1}(0)}(x_{0})$. This completes the proof. □

If we take $A=0$ and $\alpha_{n}=0$ for all $n\in\mathbb{N}\cup\{0\}$ in Theorem 3.1, then we obtain the following result.

### Corollary 3.2

*Let* *C* *be a nonempty closed convex subset of a real Hilbert space* *H*. *Let* $B: H\to2^{H}$ *be a maximal monotone operator with* $D(B)\subseteq C$. *Assume that* $(B)^{-1}(0)\neq \emptyset$. *A sequence* $\{x_{n}\}$ *generated by the following algorithm*:

$$ \textstyle\begin{cases} x_{0}\in C, \\ z_{n+1}=J_{r_{n}}(x_{n}), \\ C_{n}=\{z\in C: \Vert z_{n+1}-z \Vert \leq \Vert x_{n}-z \Vert \}, \\ Q_{n}=\{z\in C: \langle x_{n}-z,x_{0}-x_{n}\rangle\geq0\}, \\ x_{n+1}=P_{C_{n}\cap Q_{n}}(x_{0}), \end{cases} $$

*for all* $n\in\mathbb{N}\cup\{0\}$, *where* $J_{r_{n}}=(I+r_{n}B)^{-1}$ *and* $\{r_{n}\}$ *is a sequence of positive real numbers with* $0< r_{n}\leq2\alpha$ *for some* $\alpha>0$. *Then* $x_{n}\rightarrow p=P_{(B)^{-1}(0)}(x_{0})$.

## Shrinking projection methods

In this section, we introduce a new iterative shrinking projection method and prove a strong convergence theorem for finding a solution of the sum of an *α*-inverse-strongly monotone (single-value) operator and a maximal monotone (multi-valued) operator.

### Theorem 4.1

*Let* *C* *be a nonempty closed convex subset of a real Hilbert space* *H*. *Suppose that* $A: C \to H$ *is an* *α*-*inverse*-*strongly monotone operator and let* $B: H\to 2^{H}$ *be a maximal monotone operator with* $D(B)\subseteq C$ *and* $(A+B)^{-1}(0)\neq\emptyset$. *Define a sequence* $\{x_{n}\}$ *by the algorithm*

14 $$ \textstyle\begin{cases} x_{0}, z_{0}\in C_{0},\\ y_{n}=\alpha_{n}z_{n}+(1-\alpha_{n})x_{n}, \\ z_{n+1}=J_{r_{n}}(y_{n}-r_{n}Ay_{n}), \\ C_{n+1}=\{z\in C_{n}: \Vert z_{n+1}-z \Vert ^{2}\leq\alpha _{n} \Vert z_{n}-z \Vert ^{2}+(1-\alpha_{n}) \Vert x_{n}-z \Vert ^{2}\}, \\ x_{n+1}=P_{C_{n+1}}x_{0}, \end{cases} $$

*for all* $n\in\mathbb{N}\cup\{0\}$, *where* $C_{0}=C$, $J_{r_{n}}=(I+r_{n}B)^{-1}$, $\{\alpha_{n}\}$ *and* $\{r_{n}\}$ *are sequences of positive real numbers with* $0\leq\alpha_{n}\leq\beta$ *for some* $\beta\in[0,\frac{1}{2})$ *and* $0< r_{n}\leq2\alpha$. *Then the sequence* $\{x_{n}\}$ *converges strongly to a point* $p=P_{(A+B)^{-1}(0)}(x_{0})$.

### Proof

From Lemma 2.3, we see that $C_{n}$ is closed convex for every $n\in\mathbb{N}\cup\{0\}$. First, we show that $(A+B)^{-1}(0)\subset C_{n}$ for all $n\in\mathbb{N}\cup\{0\}$. For $n=0$, we have

$$(A+B)^{-1}(0)\subset C=C_{0}. $$

Suppose that $(A+B)^{-1}(0)\subset C_{k}$ for some $k\in\mathbb{N}$. Since $A: C \to H$ is an *α*-inverse-strongly monotone operator, we see that $I-r_{n}A$ is nonexpensive. Let $w \in(A+B)^{-1}(0)$. Thus $w \in C_{k}$ and

$$\Vert z_{k+1}-w \Vert ^{2}\leq\alpha_{k} \Vert z_{k}-w \Vert ^{2}+(1-\alpha_{k}) \Vert x_{k}-w \Vert ^{2}. $$

That is, $w \in C_{k+1}$. So, we have

15 $$ (A+B)^{-1}(0)\subset C_{n}, $$

for all $n\in\mathbb{N}\cup\{0\}$. It follows that $\{x_{n}\}$ is well defined.

Since $(A+B)^{-1}(0)$ is a nonempty closed convex set, there is a unique element $p\in(A+B)^{-1}(0)$ such that

$$p=P_{(A+B)^{-1}(0)}(x_{0}). $$

From $x_{n}=P_{C_{n}}(x_{0})$, we have

$$\Vert x_{n}-x_{0} \Vert \leq \Vert q-x_{0} \Vert \quad\text{for all }q\in C_{n}. $$

Due to $p\in(A+B)^{-1}(0)\subset C_{n}$, we have

16 $$ \Vert x_{n}-x_{0} \Vert \leq \Vert p-x_{0} \Vert , $$

for any $n\in\mathbb{N}\cup\{0\}$. It follows that $\{x_{n}\}$ is bounded. As $x_{n+1}\in C_{n+1}\subset C_{n}$ and $x_{n}=P_{C_{n}}(x_{0})$, we have

$$\langle x_{n}-x_{n+1},x_{0}-x_{n} \rangle\geq0, $$

for all $n\in\mathbb{N}$. This implies that

17 $$\begin{aligned} \Vert x_{n+1}-x_{n} \Vert ^{2} =& \Vert x_{n+1}- x_{0} \Vert ^{2}- \Vert x_{n}-x_{0} \Vert ^{2}-2\langle x_{n+1}-x_{n},x_{n}-x_{0} \rangle \\ \leq& \Vert x_{n+1}- x_{0} \Vert ^{2}- \Vert x_{n}-x_{0} \Vert ^{2}, \end{aligned}$$

for all $n\in\mathbb{N}$. From (16) and (17), we have

$$\begin{aligned} \sum_{n=1}^{N} \Vert x_{n+1}-x_{n} \Vert ^{2} \leq& \sum _{n=1}^{N}\bigl( \Vert x_{n+1}-x_{0} \Vert ^{2}- \Vert x_{n}-x_{0} \Vert ^{2}\bigr) \\ =& \Vert x_{N+1}-x_{0} \Vert ^{2}- \Vert x_{1}-x_{0} \Vert ^{2} \\ \leq& \Vert q-x_{0} \Vert ^{2}- \Vert x_{1}-x_{0} \Vert ^{2}. \end{aligned}$$

Since *N* is arbitrary, we see that $\sum_{n=1}^{\infty} \Vert x_{n+1}-x_{n} \Vert ^{2}$ is convergent. Thus, we have

18 $$ \Vert x_{n+1}-x_{n} \Vert \rightarrow0 \quad \text{as } n\rightarrow\infty. $$

From $x_{n+1}\in C_{n+1}$ and $\{\alpha_{n}\}\subset[0,\beta)$, it implies that

$$\begin{aligned} \Vert z_{n+1}-x_{n+1} \Vert ^{2} \leq& \alpha_{n} \Vert z_{n}-x_{n+1} \Vert ^{2}+(1-\alpha _{n}) \Vert x_{n}-x_{n+1} \Vert ^{2} \\ \leq& 2\alpha_{n} \Vert z_{n}-x_{n} \Vert ^{2}+ (1+\alpha _{n}) \Vert x_{n}-x_{n+1} \Vert ^{2} \\ \leq& 2\beta \Vert z_{n}-x_{n} \Vert ^{2}+ 2 \Vert x_{n}-x_{n+1} \Vert ^{2},\quad \forall n\in \mathbb{N}. \end{aligned}$$

By Lemma 2.2 and $\beta\in[0,\frac{1}{2})$, we obtain

19 $$ \Vert z_{n}-x_{n} \Vert \rightarrow0 \quad \text{as } n\rightarrow\infty. $$

In fact, since $\Vert z_{n+1}-x_{n} \Vert \leq \Vert z_{n+1}-x_{n+1} \Vert + \Vert x_{n+1}-x_{n} \Vert $, for all $n\in\mathbb{N}$, it follows by (18) and (19) that

20 $$ \Vert z_{n+1}-x_{n} \Vert \rightarrow0 \quad \text{as } n\rightarrow\infty. $$

Note that

$$\begin{aligned} \Vert x_{n}-y_{n} \Vert =& \bigl\Vert x_{n}-\alpha _{n}z_{n}-(1-\alpha_{n})x_{n} \bigr\Vert \\ =& \alpha_{n} \Vert x_{n}-z_{n} \Vert \\ \leq& \beta \Vert x_{n}-z_{n} \Vert , \end{aligned}$$

for all $n\in\mathbb{N}$. This implies that

21 $$ \Vert x_{n}-y_{n} \Vert \rightarrow0 \quad \text{as } n\rightarrow\infty. $$

Moreover, we note that

$$\begin{aligned}& \bigl\Vert J_{r_{n}}(I-r_{n}A)x_{n}-x_{n} \bigr\Vert \\& \quad \leq \bigl\Vert J_{r_{n}}(I-r_{n}A)x_{n}-J_{r_{n}}(I-r_{n}A)y_{n} \bigr\Vert + \bigl\Vert J_{r_{n}}(I-r_{n}A)y_{n}-z_{n+1} \bigr\Vert + \Vert z_{n+1}-x_{n} \Vert \\& \quad \leq \Vert x_{n}-y_{n} \Vert + \Vert z_{n+1}-x_{n} \Vert , \end{aligned}$$

for all $n\in\mathbb{N}$. By (20) and (21), we see that

22 $$ \bigl\Vert J_{r_{n}}(I-r_{n}A)x_{n}-x_{n} \bigr\Vert \rightarrow0 \quad \text{as } n\rightarrow\infty. $$

From (22), it follows by the demiclosed principle (see [20]) that

$$\omega_{w}(x_{n})\subset F\bigl(J_{r_{n}}(I-r_{n}A) \bigr)=(A+B)^{-1}(0). $$

By Lemma 2.1 and (16), we can conclude that the sequence $\{x_{n}\}$ converges strongly to $p=P_{(A+B)^{-1}(0)}(x_{0})$. This completes the proof. □

If we take $A=0$ and $\alpha_{n}=0$ for all $n\in\mathbb{N}\cup\{0\}$ in Theorem 4.1, then we obtain the following result.

### Corollary 4.2

*Let* *C* *be a nonempty closed convex subset of a real Hilbert space* *H*. *Let* $B: H\to2^{H}$ *be a maximal monotone operator with* $D(B)\subseteq C$. *Assume that* $(B)^{-1}(0)\neq\emptyset$. *A sequence* $\{x_{n}\}$ *generated by the following algorithm*:

$$ \textstyle\begin{cases} x_{0} \in C_{0},\\ z_{n+1}=J_{r_{n}}(x_{n}), \\ C_{n+1}=\{z\in C_{n}: \Vert z_{n+1}-z \Vert \leq \Vert x_{n}-z \Vert \}, \\ x_{n+1}=P_{C_{n+1}}x_{0}, \end{cases} $$

*for all* $n\in\mathbb{N}\cup\{0\}$, *where* $C_{0}=C$, $J_{r_{n}}=(I+r_{n}B)^{-1}$ *and* $\{r_{n}\}$ *is a sequence of positive real numbers with* $0< r_{n}\leq2\alpha$ *for some* $\alpha>0$. *Then* $x_{n}\rightarrow p=P_{(B)^{-1}(0)}(x_{0})$.

## Numerical results

In this section, we firstly follow the ideas of He *et al.* [21] and Dong *et al.* [13]. For $C=H$, we can write (4) in Theorem 3.1 as follows:

23 $$ \textstyle\begin{cases} x_{0},z_{0} \in H, \\ y_{n}=\alpha_{n}z_{n}+(1-\alpha_{n})x_{n}, \\ z_{n+1}=J_{r_{n}}(y_{n}-r_{n}Ay_{n}), \\ u_{n}=\alpha_{n}z_{n}+(1-\alpha_{n})x_{n}- z_{n+1}, \\ v_{n}= (\alpha_{n} \Vert z_{n} \Vert ^{2}+(1-\alpha_{n}) \Vert x_{n} \Vert ^{2}- \Vert z_{n+1} \Vert ^{2})/2, \\ C_{n}=\{z\in C: \langle u_{n},z \rangle\leq v_{n}\}, \\ Q_{n}=\{z\in C: \langle x_{n}-z,x_{n}-x_{0}\rangle\leq0\}, \\ x_{n+1}=p_{n},\quad\text{if }p_{n} \in Q_{n}, \\ x_{n+1}=q_{n},\quad\text{if }p_{n}\notin Q_{n}, \end{cases} $$

where

$$\begin{aligned}& p_{n} = x_{0}- \frac{\langle u_{n},x_{0}\rangle-v_{n}}{ \Vert u_{n} \Vert ^{2}}u_{n}, \\& q_{n} = \biggl( 1-\frac{\langle x_{0}-x_{n}, x_{n}-p_{n}\rangle }{\langle x_{0}-x_{n}, w_{n}-p_{n}\rangle} \biggr)p_{n}+ \frac{\langle x_{0}-x_{n}, x_{n}-p_{n}\rangle}{\langle x_{0}-x_{n}, w_{n}-p_{n}\rangle}w_{n}, \\& w_{n} = x_{n}- \frac{\langle u_{n},x_{n}\rangle- v_{n}}{ \Vert u_{n} \Vert ^{2}}. \end{aligned}$$

Let $R^{2}$ be the two dimensional Euclidean space with usual inner product $\langle x,y\rangle=x_{1}y_{1}+x_{2}y_{2}$ for all $x=(x_{1},x_{2})^{T}$, $y=(y_{1},y_{2})^{T}\in R^{2}$ and denote $\Vert x \Vert =\sqrt{{x_{1}}^{2}+{x_{2}}^{2}}$.

Define the operator $A': R^{2}\to R^{2}$ as

$$A'(x)=\biggl(0, \frac{1}{2}x_{1} \biggr)^{T }\quad \text{for all } x=(x_{1},x_{2})\in R^{2}. $$

It is obvious that $A'$ is nonexpansive and hence $I-A'$ is $\frac {1}{2}$-inverse-strongly monotone (see [17, 22]). Thus we have the mapping $A=I-A': R^{2}\to R^{2}$ as

$$A(x)=\biggl(x_{1},x_{2}-\frac{1}{2}x_{1} \biggr)^{T }\quad \text{for all } x=(x_{1},x_{2})\in R^{2} $$

is $\frac{1}{2}$-inverse-strongly monotone. Let $W=\{(x_{1},x_{2})\in R^{2}: x_{1}=x_{2}\}$. Then *W* is a linear subspace of $R^{2}$. Define

$$N_{W}=\bigl\{ (x,y): x \in W \text{ and } y\in W^{\perp} \bigr\} . $$

This implies that $N_{W}$ is maximal monotone (see [23]). It is easily seen that $(A+N_{W})^{-1}(0)\neq\emptyset$. We take $\{r^{(n)}\}=\{\frac {1}{n+2}\}\subset(0,1)$ (note $\alpha= \frac{1}{2}$). Then $\{r^{(n)}\}$ is a sequence of positive real numbers in $(0,2\alpha)$, and $\{\alpha^{(n)}\}=0.1$ (note $\beta= 0.4$). Let $x^{(0)}=(4,3), (-2,8), (3,-4)$ and $(-1,-3)$ be the initial points and fixed $z^{(0)}=(1,1)$. Denote

$$E(x)=\frac{ \Vert x^{(n)}-J_{r^{(n)}}(x^{(n)}-r^{(n)}Ax^{(n)}) \Vert }{ \Vert x^{(n)} \Vert }. $$

Since we do not know the exact value of the projection of $x_{0}$ onto the set of fixed points of $J_{r_{n}}(I-r_{n}A)$, we take $E(x)$ to be the relative rate of convergence of our algorithm. In the numerical result, $E(x)<\varepsilon $ is the stopping condition and $\varepsilon= 10^{-7}$. Moreover, we have shown that the competitive efficacy of our example, see Table 1.

**Table 1:** **This table illustrates that in our examples (** **23** **) derived from (** **4** **) has a competitive efficacy**

$\boldsymbol{x^{(0)}}$	**Iter.**	$\boldsymbol{x=(x_{1},x_{2})^{T}}$	***E*** **(** ***x*** **)**
(4,3)	4520	(1.573198640818142,1.573198530023523)	3.521317011074167*e* − 08
(−2,8)	5420	(0.944819548758385,0.944819526356611)	1.185505467234501*e* − 08
(3,−4)	3307	(99.631392375764780,99.631402116509490)	4.888391102078766*e* − 08
(−1,−3)	4110	(−0.781555402714756,−0.781555394005797)	5.571556279844247*e* − 09

## Conclusions

We have proposed two new iterative algorithms for finding the common solution of the sum of two monotone operators by using hybrid methods and shrinking projection methods. The convergence of the proposed algorithms is obtained and the numerical result of the hybrid iterative algorithm is also effective.

## References

[1] Attouch H, Thera M (1996). A general duality principle for the sum of two operators. J. Convex Anal..

[2] Bauschke HH (2009). A note on the paper by Eckstein and Svaiter on general projective splitting methods for sums of maximal monotone operators. SIAM J. Control Optim..

[3] Chen YQ, Cho YJ, Kumam P (2016). On the maximality of sums of two maximal monotone. J. Math. Anal..

[4] Chen GHG, Rockafellar RT (1997). Convergence rates in forward-backward splitting. SIAM J. Optim..

[5] Cho SY, Qin X, Wang L (2014). A strong convergence theorem for solutions of zero point problems and fixed point problems. Bull. Iran. Math. Soc..

[6] Lions PL, Mercier B (1979). Splitting algorithms for the sum of two nonlinear operators. SIAM J. Numer. Anal..

[7] Mahey P, Pham DT (1993). Partial regularization of the sum of two maximal monotone operators. RAIRO Model. Math. Anal. Num..

[8] Passty GB (1979). Ergodic convergence to a zero of the sum of monotone operator in Hilbert spaces. J. Math. Anal. Appl..

[9] Svaiter BF (2011). On Weak Convergence of the Douglas-Rachford Method. SIAM J. Control Optim..

[10] Moudafi A, Thera M (1997). Finding a zero of the sum of two maximal monotone operators. J. Optim. Theory Appl..

[11] Nakajo K, Takahashi W (2003). Strong convergence theorems for nonexpansive mappings and nonexpansive semigroups. J. Math. Anal. Appl..

[12] Saewan S, Kumam P (2015). Computational of generalized projection method for maximal monotone operator and a countable family of relatively quasi-nonexpansive mappings. Optimization.

[13] Dong QL, Lu YY (2015). A new hybrid algorithm for a nonexpansive mapping. Fixed Point Theory Appl..

[14] Takahashi W, Takeuchi Y, Kubota R (2008). Strong convergence theorems by hybrid methods for families of nonexpansive mappings in Hilbert spaces. J. Math. Anal. Appl..

[15] Takahashi W (2009). Introduction to Nonlinear and Conex Analysis.

[16] Kamimura S, Takahashi W (2000). Approximating solutions of maximal monotone operators in Hilbert spaces. J. Approx. Theory.

[17] Takahashi W (2000). Nonlinear Functional Analysis.

[18] Matinez-Yanes C, Xu HK (2006). Strong convergence of the CQ method for fixed point processes. Nonlinear Anal..

[19] Aoyama K, Kimura Y, Takahashi W, Toyoda M (2007). On a strongly nonexpansive sequence in Hilbert spaces. J. Nonlinear Convex Anal..

[20] Goebel K, Kirk WA (1990). Topics in Metric Fixed point Theory.

[21] He S, Yang C, Duan P (2010). Realization of the hybrid method for Mann iterations. Appl. Math. Comput..

[22] Browder FE, Petryshyn WV (1967). Construction of fixed points of nonlinear mappings in Hilbert space. J. Math. Anal. Appl..

[23] Eckstein J, Bertsekas DP (1992). On the Douglas-Rachford splitting method and the proximal point algorithm for maximal monotone operators. Math. Program..

